# Pressure sore at an unusual site: the bilateral knee – a case report

**DOI:** 10.1097/MS9.0000000000000993

**Published:** 2023-06-17

**Authors:** Pawan Acharya, Bipin Poudel, Shubham Shrestha, Bunu Maharjan

**Affiliations:** a Department of Plastic Surgery, Lister Hospital, Stevenage, United Kingdom; b Department of Surgery, Patan Academy of Health Sciences; c Lalitpur Nursing Campus, Lalitpur, Nepal

**Keywords:** case report, knee, pressure sore

## Abstract

A pressure ulcer is a skin and soft tissue injury that commonly occur on bony prominences like the ischium, sacrum, heel, malleolus, and occiput but the knee is not the usual site for pressure ulcers. Here, the authors present a case of a pressure ulcer over an unusual site, the knee.

**Case presentation:**

The authors reported a case of a 66-year-old male, last seen 5 days ago by his son, who was found on the floor (with the knee on the ground) and was brought to the hospital. The patient had no history of mobility issue. On initial assessment his vitals were unstable but Glasgow Coma Scale 15/15 and CT head and ECG were unremarkable. On knee examination, there was bilateral grazing and bruising diagnosed as grade 3 and grade 4 pressure sore in the right and left knee, respectively. The pressure ulcer was managed adhering to the principles to remove all pressure, keep the ulcer clean, prevent further injury, and regular dressing by tissue viability nurses. On 17 March 2023, the patient was discharged from the hospital to a care home after his condition improved.

**Clinical discussion:**

A comprehensive review of the medical literature found no other reports of pressure sore at knee. A few published articles showed pressure sore as a complication of prone positioning. It is postulated that fall and long-term lie on the knees have developed this pressure ulcer.

**Conclusion:**

The Clinicians should be vigilant to check for pressure ulcers especially in all the bony prominences in any patients having an unwitnessed fall.

## Introduction

Highlights

- This is the first case reported of pressure sore at the knee.
- Pressure sore commonly occur on bony prominences like the ischium, sacrum, heel, malleolus, and occiput.
- The longer length of time spent in the same position without pressure relief leads to pressure sore.
- We should be vigilant to check for pressure ulcers especially in the bony prominences in any patients having an unwitnessed fall.

A pressure ulcer is a skin and soft tissue injury that form because of constant or prolonged pressure exerted on the skin. Pressure ulcers most commonly occur over bony prominences such as the ischium, greater trochanter, sacrum, heel, malleolus (lateral than medial), and occiput but can develop on any part of the body^1^. Local factors such as long sustained local pressure or a short period of high pressure, shearing forces, friction, and moisture while sitting or lying likely lead to the development of pressure ulcers^2^. The combination of impairment due to immobility, nutritional deficiency, and chronic diseases involving multiple systems predispose the elderly population to high risk^3^. Though typical ulcers occur over bony prominences, the knee is not the usual site for pressure ulcers. Here, we present a case of a pressure ulcer over an unusual site, the knee. The work has been reported in line with the Surgical CAse REport (SCARE) 2020 criteria^4^.

## Case report

A 66-year-old male, last seen 5 days ago by his son, was found on the floor (with the knee on the ground) by the son as he did not hear from him for a few days. The son called the ambulance crew, who brought the patient to Accident and Emergency (A and E) on 26 January 2023. The patient remembered falling and hitting his head but was unsure of the exact day or time. He denied any recent illness and mentioned that he had been as per his baseline. However, he did mention that he has had episodes of occasional drowsiness.

The patient lived by himself in a house and had no mobility issues. He was an ex-smoker and had stopped cigarettes 20 years ago. He drank greater than 14 units of alcohol per week. He had been diagnosed with atrial fibrillation 14 years ago but was not taking any regular medications.

**Figure 1:**
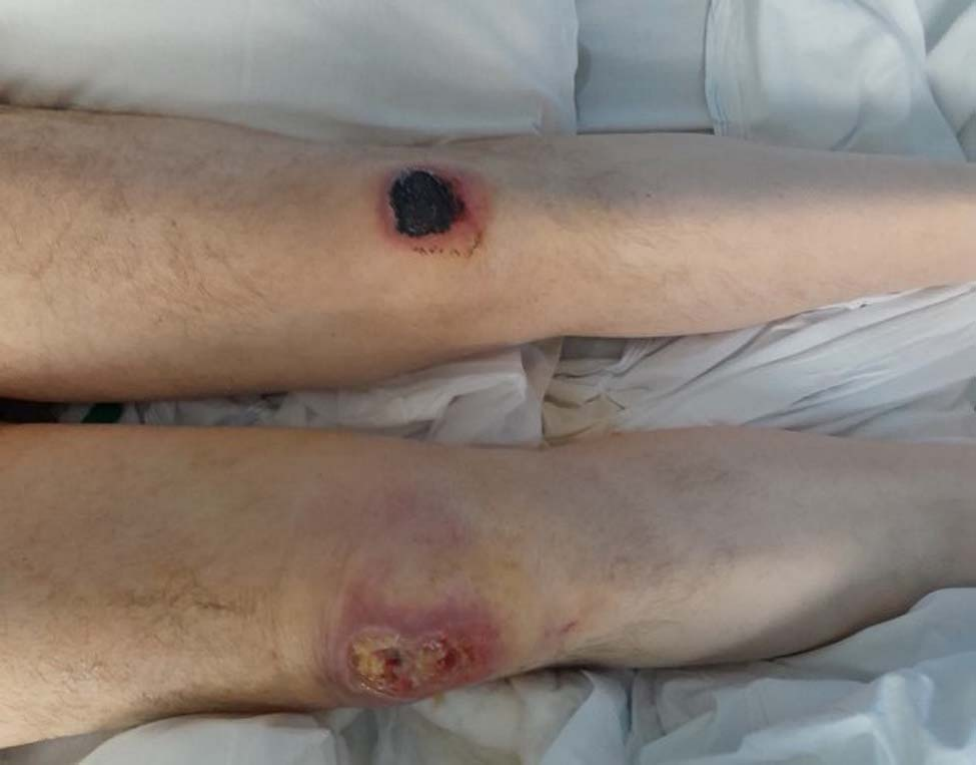
Bilateral pressure sore over knee.

On initial assessment in A and E, he was alert but occasionally drowsy, and in respiratory distress. He has had multiple bruises on B/L knees. His vitals were unstable with a SpO_2_ of 94% maintained in 4 l O_2_, BP 100/70 mmHg, and heart rate of 110 beats per minute. His Glasgow Coma Scale was 15/15 and his CT head and ECG were unremarkable. On knee examination, there was bilateral grazing and bruising as shown in the Figure 1. Blood investigation showed the patient had AKI due to rhabdomyolysis secondary to long lie and sepsis, so he was admitted to intensive therapy unit on 26 January 2023 for more than a month where he was treated with IV antibiotics and underwent dialysis. He was stepped down from intensive therapy unit and admitted to the renal ward on 03 March 2023.

**Figure 2:**
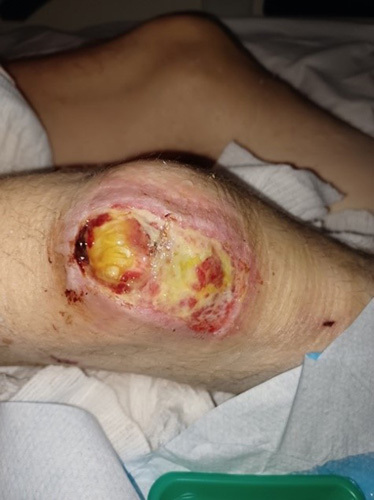
Pressure ulcer over right knee.

**Figure 3:**
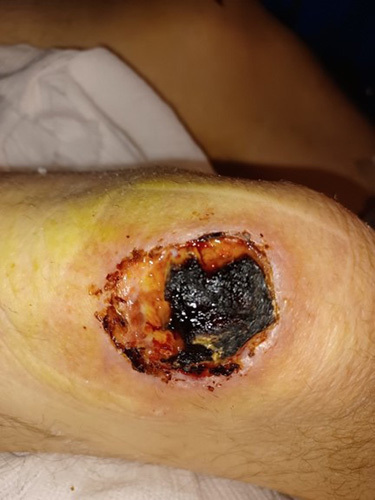
Pressure ulcer over left knee.

Bruising in the bilateral knee was referred to plastic surgery which was diagnosed as a grade 3 and grade 4 pressure sore in the right and left knee, respectively (Figs. 2 and 3). The pressure ulcer was managed by plastic surgeon and the team adhering to the principles to remove all pressure, keep the ulcer clean, prevent further injury, and regular dressing by tissue viability nurses.

On 17 March 2023 the patient was discharged from the hospital to a care home after his condition improved. His pressure ulcer has been healing well with healthy granulation tissue around the wound site. Community nurses were involved in regular dressing changes for pressure ulcers. The patient believes the excessive intake of alcohol led to this condition.

## Discussion

Pressure ulcers typically occur over a bony prominence and the majority of ulcers are located in the low back area, including the sacrum, coccyx, ischium, buttocks, greater trochanters, and the leg area including the heels and lateral malleoli^1^. These locations account for 87% of all pressure ulcers^5^.

A comprehensive review of the medical literature found no other reports of pressure sore at the knee. We searched PubMed articles and Google Scholar articles with the headings decubitus ulcer or pressure sore or pressure ulcer or bed sore and knee. Reference lists from related articles were also manually searched.

A few published articles showed pressure sore as a complication of prone positioning. Prone positioning for 5 days resulted in the bilateral necrosis of the papilla mammae in a male patient as described in a case report^6^. In prone position forehead, chin, shoulders, thorax, pelvis, knees, and ankles are showed to be as weight bearing surface as per study of the effect of patient positioning by Curley^7^. Moreover, as the skin in this areas is not accustomed to weight bearing so unusual patient positioning in a prone position will increase risk of pressure sore.

In the patient described here, multiple factors could have likely to contribute to development of pressure ulcers. The length of time spent in the same position without pressure relief was likely the largest contributing factor. He spent 5 days in this position before his son finally found him and rushed him to the hospital. Decreased local tissue oxygenation must be the probable cause to contribute to pressure ulcer development at the knee.

A typical pressure sores were mostly seen in patients either due to the use of medical devices, increased spasticity (contracture), or bone deformity^8^, however, this is not the case here in this fairly healthy man.

Thereby, it is postulated that fall and long-term lie on the knees have developed this pressure ulcer.

## Conclusion

Pressure ulcers in subdermal tissues under bony prominences very likely occur between the first hour and 4–6 h after sustained loading^9^. We should be vigilant to check for pressure ulcers especially in the bony prominences in any patients having an unwitnessed fall. Pressure ulcer can also occur in unusual location. We report the first case of a bilateral knee pressure ulcer.

## Ethical approval

None. Since this report involves no experiments, the ethical approval is waived.

## Consent

Written informed consent was obtained from the patient for publication of case report. A copy of the written consent is available for review by the Editor-in-Chief of this journal on request.

## Sources of funding

None.

## Author Contribution

P.A. is involved in conceptualization. All authors P.A., B.P., S.S., and B.M. have contributed to the writing, editing, and preparation of the manuscript and have reviewed it before submission.

## Conflicts of interest disclosures

The authors declare that they have no financial conflict of interest with regard to the content of this report.

## Research registration unique identifying number (UIN)

1. Name of the registry: N/A.
2. Unique Identifying number or registration ID: N/A.
3. Hyperlink to your specific registration (must be publicly accessible and will be checked): N/A.

## Guarantor

Pawan Acharya.

## Provenance and peer review

Not commissioned, externally peer- reviewed.
